# Inhaled granulocyte-macrophage colony stimulating factor for mild-to-moderate autoimmune pulmonary alveolar proteinosis - a six month phase II randomized study with 24 months of follow-up

**DOI:** 10.1186/s13023-020-01450-4

**Published:** 2020-07-02

**Authors:** Xinlun Tian, Yanli Yang, Lulu Chen, Xin Sui, Wenshuai Xu, Xue Li, Xiaobei Guo, Lingshan Liu, Yusen Situ, Jun Wang, Yang Zhao, Shuzhen Meng, Wei Song, Yonglong Xiao, Kai-Feng Xu

**Affiliations:** 1 Department of Pulmonary and Critical Care Medicine, Peking Union Medical College Hospital, Chinese Academy of Medical Sciences & Peking Union Medical College, Beijing, China; 2 Department of Pulmonary and Critical Care Medicine, The Affiliated Drum Tower Hospital of Nanjing University Medical School, Jiangsu, China; 3 Department of Radiology, Peking Union Medical College Hospital, Chinese Academy of Medical Sciences & Peking Union Medical College, Beijing, China; 4 Emergency Center, Beijing Tongren Hospital, Capital Medical University, Beijing, China; 5 Department of Biochemistry, Faculty of Medicine and Dentistry, University of Alberta, Edmonton, Canada

**Keywords:** Autoimmune pulmonary alveolar proteinosis, Granulocyte-macrophage colony stimulating factor, Inhalation

## Abstract

**Background:**

Treatment of autoimmune pulmonary alveolar proteinosis (aPAP) by inhaled granulocyte-macrophage colony stimulating factor (GM-CSF) is considered safe and effective. Evidence of benefit from GM-CSG inhalation for mild to moderate aPAP patients is limited.

**Methods:**

In this multicenter, randomized, open-labeled clinical trial, 36 aPAP patients with mild to moderate disease severity were randomized into either the GM-CSF treatment group or control group. Inhaled GM-CSF was prescribed for 6 months, and patients received follow-up for another 18 months without treatment. Physiological features of the patients were analyzed.

**Results:**

There were 36 patients (19 in the treatment group, 17 in the control group) included. There were no significant differences in the primary endpoints as measured by the change of alveolar arterial oxygen gradient (A-aDO_2_) from the baseline values to the values obtained during treatment or during the following 18-month non-treatment observation period [control group vs. treatment group: 0.51 ± 12.09 mmHg vs. -0.35 ± 13.76 mmHg, *p* = 0.848 (3 month); 1.85 ± 11.21 mmHg vs. 7.31 ± 8.81 mmHg, *p* = 0.146 (6 months); 6.05 ± 11.14 mmHg vs. 6.61 ± 10.64 mmHg, *p* = 0.899 (24 months)]). Percentage of diffusion capacity predicted (DLCO%) and percentage of total lung capacity predicted (TLC%), however, were significantly improved in the treatment group by the end of the study (*P* = 0.010 and 0.027). St. George Respiratory questionnaire (SGRQ) scores were better after 6 months treatment with GM-CSF compared to the control group, and the benefits of treatment were maintained throughout the observation period. No severe side effects were observed during the study.

**Conclusion:**

Six months of inhaled GM-CSF treatment had no effect on the alveolar–arterial oxygen gradient in patients with mild to moderate pulmonary alveolar proteinosis. There were changes in some clinical or laboratory measures, but no clinically important changes were noted at the end of study. (Clinical Trial Registry: NCT02243228, Registered on September 17, 2014, https://www.clinicaltrials.gov/ct2/show/NCT02243228?term=NCT02243228&draw=2&rank=1)

## Background

Autoimmune pulmonary alveolar proteinosis (aPAP, previously known as idiopathic PAP) is a rare interstitial lung disease elicited by the formation of autoantibodies which neutralize the activity of granulocyte-macrophage colony stimulating factor (GM-CSF), consequently decreasing macrophage clearance of surfactant [1]. Currently, the standard treatment strategy for PAP is whole lung lavage (WLL). About 70% patients need another WLL within 3 years due to recurrence [2, 3]. Patients who undergo WLL require general anesthesia and double-lumen endotracheal intubation, which means only hospitals with experienced physicians can perform the procedure. Considering the recurrence rate and the cumbersome procedure of WLL, whether or not patients with mild or moderate disease should obtain that treatment is a matter of controversy.

Inhaled GM-CSF therapy has become an attractive alternative option for aPAP patients not only due to its effectiveness and safety [4, 5], but also because it is a convenient treatment method for patients who are reluctant to receive WLL. Previous studies included small sample sizes, and as a result, disease severity has not been stratified. Nevertheless, whether patients with mild or moderate disease will benefit from GM-CSF treatment over the long term is still unclear. We prospectively evaluated if inhaled GM-CSF would delay disease progression in patients with mild-to moderate aPAP over a two-year period.

## Methods

### Participants

Patients with mild or moderate aPAP, aged between 18 and 80 years old, were enrolled at two hospitals, including Peking Union Medical College Hospital (PUMCH) and The Affiliated Drum Tower Hospital of Nanjing University Medical School in China.

The inclusion criteria included: (1) patients with a clinical diagnosis of PAP by high-resolution computed tomography (HRCT), further pathologically confirmed by testing for amorphous periodic Acid-Schiff (PAS)-positive granules; (2) a positive serum GM-CSF antibody test which indicated an elevated serum GM-CSF antibody level; and (3) patients eligible for the trial should have progressive or unremitting PAP, defined as worsening or unchanging PaO_2_ or A–aDO_2_ over a 3-month period of observation. PAS-positive granules were found either in milky broncho-alveolar lavage fluid (BALF) or in alveolar structures of lung biopsy tissues which were obtained as follows: cytological findings of bronchial lavage fluid only (BAL) (*n* = 13), transbronchial lung biopsy only (TBLB) (*n* = 7), both TBLB and BAL (*n* = 12), percutaneous lung puncture biopsy (*n* = 3), and surgical lung biopsy only (n = 1). The GM-CSF antibody test was performed according to the method established by Uchida et al. [6, 7]. Our hospital set the cutoff point at 2.39 g/mL, with measurements in excess of this value resulting a positive report [8].

Disease severity was assessed with a disease severity score (DSS), with patients with a DSS of 1 to 3 inclusive being included in our study. DSS scores were defined by Inoue et al. as follows [3]: Grade 1: No symptoms and an arterial oxygen partial pressure (PaO_2_) ≥ 70 mmHg; Grade 2: PaO_2_ ≥ 70 mmHg with symptoms; Grade 3: PaO_2_ between 60 and 70 mmHg; Grade 4: PaO_2_ between 50 and 60 mmHg; and Grade 5: PaO_2_ below 50 mmHg.

Individuals were excluded if they met the following criteria: (1) patients had already received previous GM-CSF therapy or other forms of cytokine therapy, or had undergone lung lavage therapy within the 3 months prior to enrollment; (2) Individuals with PAP resulting from other conditions (e.g. occupational exposure to silica, underlying human immunodeficiency virus infection, respiratory infections, myeloproliferative disorder or leukemia); (3) Individuals with histories of severe allergic or anaphylactic reactions to humanized or murine monoclonal antibodies; (4) Individuals with chronic lung diseases or any other serious medical conditions, or (5) Women who were pregnant, lactating or planned to become pregnant during the study period.

### Study design

**Fig. 1:**
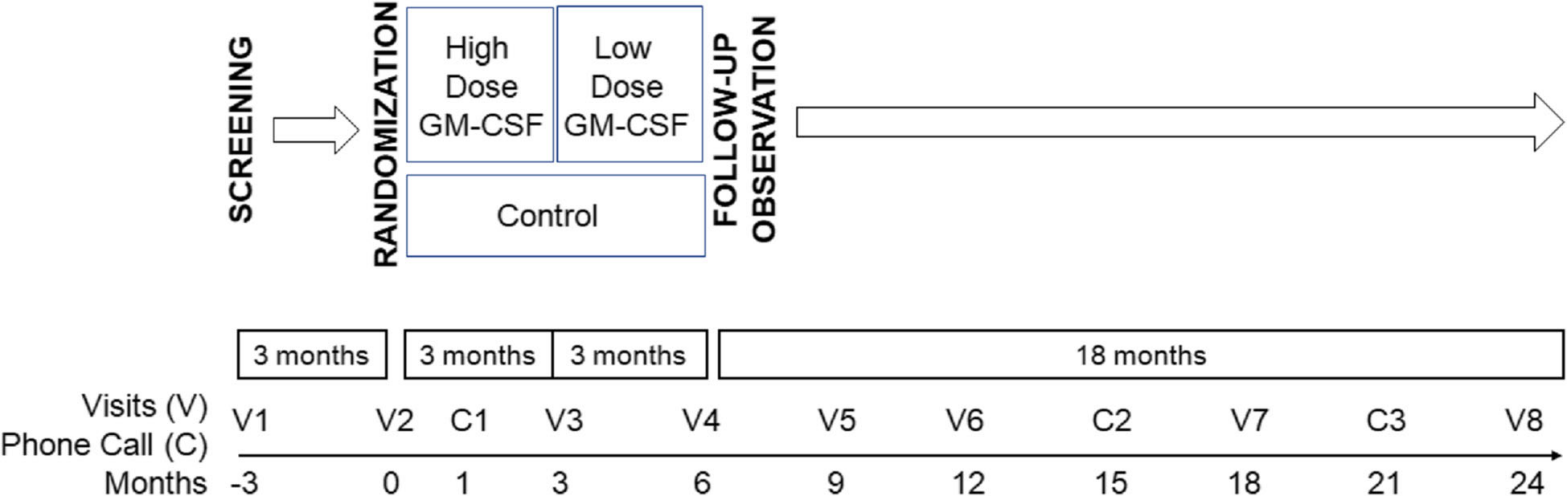
The clinical trial comprised of three sequential periods: high-dose therapy for 3 months (150 μg twice daily every other week), low-dose therapy for 3 months (150 μg once daily every other week) and observation for 18 months. Study visits during treatment were designed at 0, 3 and 6 months. Thereafter, patients were followed up with using visits at 9, 12, 18 and 24 months. Patients’ safety questionnaires were reviewed by telephone at 1. 15 and 21 months

This was a multicenter, randomized, open-label clinical trial (clinical trial number: NCT02243228, Inhalation of granulocyte-macrophage colony stimulating factor for autoimmune pulmonary alveolar proteinosis) comprising three sequential periods: high-dose therapy for 3 months, low-dose therapy for 3 months and observation for 18 months. Study visits during treatment were designed at 0, 1, 3 and 6 months. Thereafter, patients were followed up by visits at 9, 12, 15, 18, 21 and 24 months (Fig. 1). Patients’ safety questionnaires were reviewed by telephone at 1, 15 and 21 months. Before the therapeutic trial, all participants entered an initial 3-month observation period, during which disease severity and progression were evaluated. Participants that had their PaO_2_ increase by 10 mmHg or more, or alveolar-arterial oxygen gradient (A–aDO_2_) decrease by 10 mmHg or more were regarded as having undergone spontaneous improvement and were excluded from enrollment. It should be noted that if a participant was acquainted to the principal investigator as a patient with a well-documented history showing an unremitting aPAP state, he/she could be enrolled into the study without this observation period. After 3-months of observation, all unremitting PAP patients underwent a stratified randomization based on their DSS at the time of enrollment to ensure equal representation of patients with various disease severities in both the treatment group and the placebo group using a random number table. The randomization was blinded to both the patients and the investigators.

Recombinant human GM-CSF (rhGM-CSF) was administered to patients in treatment group by inhalation as previously described [9]. 150 μg of rhGM-CSF was dissolved in 2 ml of sterile saline, and was inhaled as an aqueous aerosol using an LC-PLUS nebulizer with a manual interrupter valve connected to a PARI Turbo BOY compressor (PARI GmbH, Starnberg, Germany) [10]. Treatment was designed according to a previous study [11], including 3 months of high-dose GM-CSF administration (150 μg twice daily every other week) and another 3 months of low-dose administration (150 μg once daily every other week), serving as induction and maintenance therapy, respectively.

In the control group, patients were not prescribed any kind of treatment related to PAP (including GM-CSF, WLL or anti-CD20, etc.) but had the same follow up plan as the treatment group.

In previous published studies, patients inhaling GM-CSF had a mean change in A–aDO_2_ of 11 mmHg [11]. Thus, the target sample size was 25, chosen to give a detection power of 90%, allowing for a 5% incidence of type-I error. After taking other outcome measurements and participant dropout into consideration, the study size was increased to 35–45 patients.

The study was approved by the institutional review board of PUMCH (Approval No. S-717) and reviewed by the institutional review boards at the Affiliated Drum Tower Hospital of Nanjing University Medical School. All participants provided informed written consent prior to enrollment.

### Assessments

Clinical information including history, symptoms, serological (including lactate dehydrogenase, carcinoembryonic antigen levels) and radiological features, pulmonary function testing results and physical examination results were obtained at each visit during the study. Arterial blood gas analysis (ABG) tests were performed with patients that had been breathing room air for at least 15 min. A low dose quantitative computed tomography of the chest (in PUMCH) or high resolution CT (HRCT) of the chest (in The Affiliated Drum Tower Hospital of Nanjing University Medical School) was obtained before and after GM-CSF therapy and evaluated in a blinded fashion by a board-certified radiologist. The original CT measurements were collected, and the total lung volume and mean lung density were automatically calculated and post-processed with Pulmo 3D (syngo. Via, version VA 30, Siemens Healthcare, Germany) for the automatic segmentation of the pulmonary parenchyma by excluding the intrapulmonary vessels following the process published by one of our co-authors, Dr. Sui [12].

Intergroup differences in the change of A–aDO_2_ from baseline to the end of treatment were defined as primary endpoints.

Other data, representing the efficacy of GM-CSF inhalation, were also evaluated as secondary endpoints, including pulmonary function test differences between the treatment group and the control group (forced vital capacity [FVC], total lung capacity [TLC], diffusing capacity for carbon monoxide [DLCO] or diffusing capacity for carbon monoxide corrected for alveolar volume [DLCO/VA]), 6 min walking distance differences between the groups, and relapse time in the two groups. The definition of relapse was as follows: 1) a new requirement for whole lung lavage (WLL) or any other kind of treatment (including traditional medicine, subcutaneous injection or GM-CSF inhalation) due to disease progression; or 2) PAP death; or 3) reduction in PaO_2_ of more than 10 mmHg, or increase in A–aDO_2_ of more than 10 mmHg; or 4) a worsened chest HRCT independently confirmed by two physicians. Monitoring for adverse events was conducted during the study, looking for airway hypersensitivity, fever, mylagia, arrhythmia and potential effects on the circulatory system.

All blood tests were performed in laboratories affiliated with the two hospitals, both of which have China’s quality management certification. Serum levels of GM-CSF antibody were tested at PUMCH.

All of the data was collected and stored in the database system founded by Beijing Yikang Healthcare Technology Co.

### Statistical analysis

All statistical analyses were performed using SPSS 20.0 software. Numeric results were presented as either the mean ± SD or the median and inter-quartile range. Metric variables were shown as the mean and categorical variables were given in terms of frequencies and percentages. The *X*^2^ test was used to analyze proportions of variables. For group comparisons, the unpaired *t* tests and *Wilcoxon* rank-sum test were used to evaluate the differences in normally distributed variables. *Kaplan-Meier* Curve analysis was used to analyze time for relapse in the two groups. All *P* values reported were two-sided.

## Results

### Baseline demographic information

**Fig. 2:**
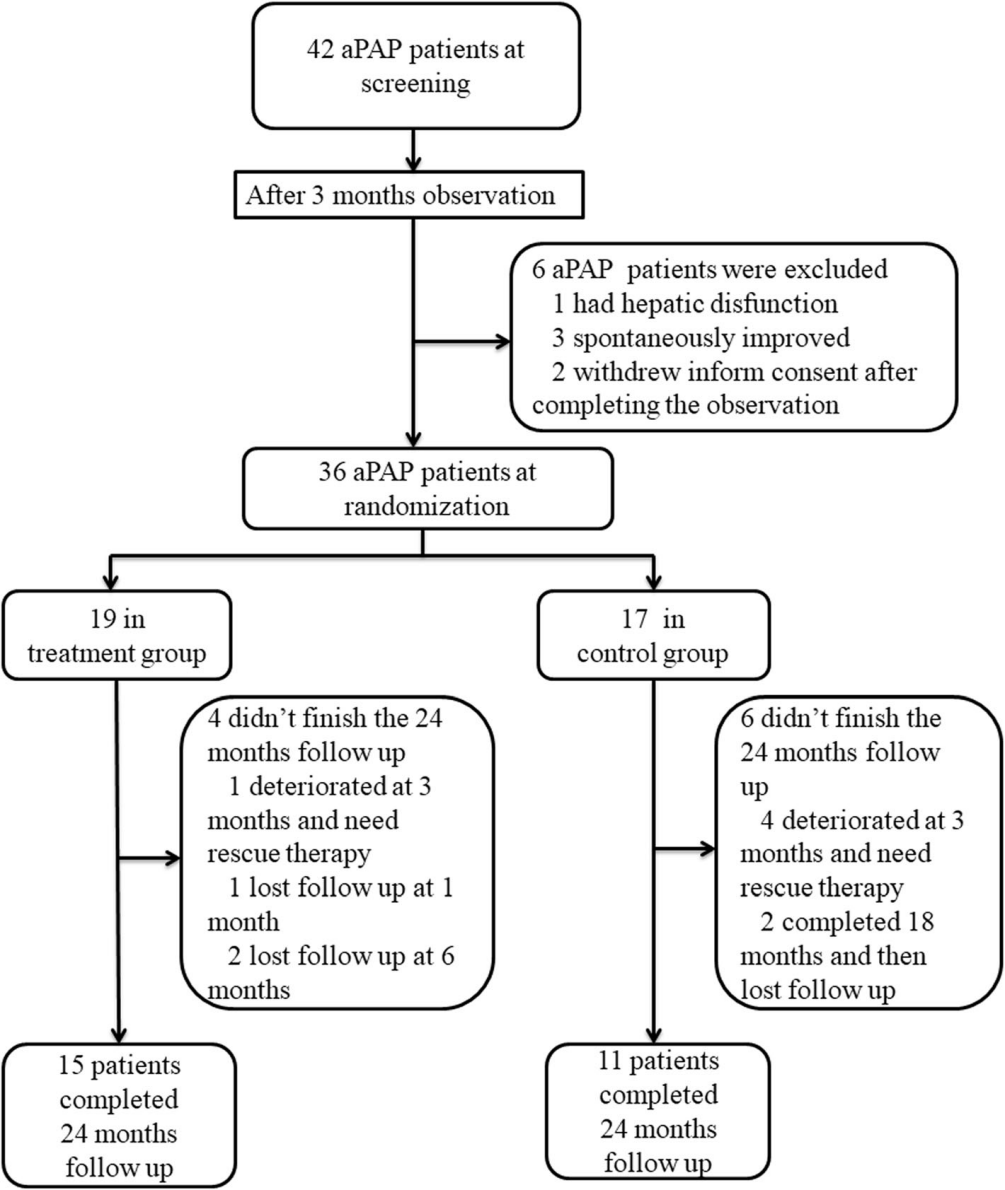
Flow diagram of the study cohort. aPAP: autoimmune pulmonary alveolar proteinosis; GM-CSF: granulocyte-macrophage colony stimulating factor

Forty-two aPAP patients were screened and 36 patients were randomized (19 in the treatment group and 17 in the control group). After 24 months of follow up, 26 patients (72.2%, 15 from the treatment group and 11 from the control group) completed the study. The period of recruitment and follow up was from July 20, 2014 to July 6, 2018 after the last enrolled patient completed his 24 months follow up. In the treatment group, one patient deteriorated at 3 months and required rescue therapy (WLL). Another patient lost follow up at 1 month and two more patients withdrew at 6 months. In the control group, 4 patients deteriorated at 3 months and required rescue therapy (one received GM-CSF inhalation, two received WLL and one was prescribed traditional medicine). 2 patients withdrew at 21 months. (Fig. 2).

In 36 patients, the most common presenting symptom was dyspnea (20/36, 55.6%), followed by cough (13/36, 36.1%), phlegm (6/36, 16.7%) and chest pain (3/36, 8.3%). 4 out of our 36 patients were diagnosed by regular health check-up without any symptoms. The median duration of symptoms is 6 months (inter-quartile range is from 0 to 60 months) in our patients. All of our patients had extent bilateral pulmonary infiltrates confirmed by HRCT.

Demographic information of the 36 patients entered the study is shown in Table 1. There were no significant differences in demographic information between the treatment group and the control group including age and sex. No significant differences were found in patients’ disease severity markers at baseline, including symptoms, ABG, pulmonary function tests, 6 min walking distance (6MWD) and anti-GM-CSF antibody levels between the two groups.

**Table 1:** Demographic features of autoimmune pulmonary alveolar proteinosis (aPAP) patients at baseline

Parameter		Control group (*n* = 17)	Treatment group (*n* = 19)	*P* value
Age (year)		42.88 ± 12.75	43.53 ± 12.89	0.881
Sex (female/male)		4/13	6/13	0.717
Duration of the disease (months)^a^		6 (6–60)	6 (0.5–60)	0.852
Smoking status	Never	7	7	0.965
	Ex-smoker	5	6	
	Current smoker	5	6	
Disease Severity Score	1	0	0	0.409
	2	13	11	
	3	4	8	
Hb (g/dL)		16.11 ± 1.61	16.00 ± 1.64	0.839
HCT (%)		46.07 ± 3.7	45.74 ± 4.47	0.810
LDH (U/L)		244.06 ± 53.02	233.82 ± 43.43	0.547
CEA (U/L)		5.34 ± 4.96	4.74 ± 3.41	0.678
FEV_1_ pred (%)		78.09 ± 14.12	79.16 ± 15.68	0.832
FVC pred (%)		79.14 ± 13.42	79.71 ± 13.70	0.900
TLC pred (%)		74.59 ± 9.65	74.82 ± 10.78	0.946
DLCO pred (%)		69.50 ± 13.94	68.41 ± 16.90	0.835
DLCO/VA pred (%)		98.58 ± 18.45	98.67 ± 24.32	0.990
PaO_2_ (mmHg)		77.51 ± 8.53	76.88 ± 11.23	0.854
A-aDO_2_ (mmHg)		28.32 ± 9.09	28.74 ± 11.04	0.902
SGRQ symptom		24.06 ± 13.55	29.17 ± 29.75	0.506
SGRQ activity		30.70 ± 18.46	30.33 ± 16.55	0.949
SGRQ effect		24.20 ± 16.10	21.83 ± 21.29	0.712
SGRQ total		26.47 ± 14.74	27.38 ± 19.75	0.878
6MWD		495.25 ± 79.39	477.95 ± 65.68	0.485
SpO_2_ at the end of 6MWD		94.87 ± 2.70	94.58 ± 5.20	0.847
Mean lung density		− 718.62 ± 82.70	− 687.25 ± 68.48	0.315
Total lung volume radiological measurement (ml)		4556.08 ± 841.15	4461.08 ± 1399.60	0.841
GM-CSF antibody (**μ** g/ml)		75.86 ± 93.94	73.30 ± 58.65	0.922
Treatment before the trial	Never	13	14	0.489
	WLL	3	5	
	Others^b^	1	0	

*Abbreviations*: *aPAP* autoimmune pulmonary alveolar proteinosis, *A-aO*_*2*_ alveolar arterial oxygen gradient, *CEA* carcinoembryonic antigen, DLCO: DLCO_:_ diffusing capacity for carbon monoxide, *DLCO/VA* diffusing capacity for carbon monoxide corrected for alveolar volume, *FEV1* forced expiratory volume in the first second, *FVC* forced vital capacity, *GM-CSF* granulocyte macrophage colony stimulating factor, *Hb* hemoglobin, *HCT* hematocrit, *LDH* lactate dehydrogenase, *PaO*_*2*_ partial pressure of oxygen, *SGRQ* St George Respiratory Questionnaire, *SpO*_*2*_ oxygen saturation in pulse oximetry, *TLC* total lung capacity, *6MWD* 6 min walking distance (test)

^a^: median (inter-quartile range)

^b^:Traditional medicine treatment

### Primary endpoint: A-aDO_2_

**Fig. 3:**
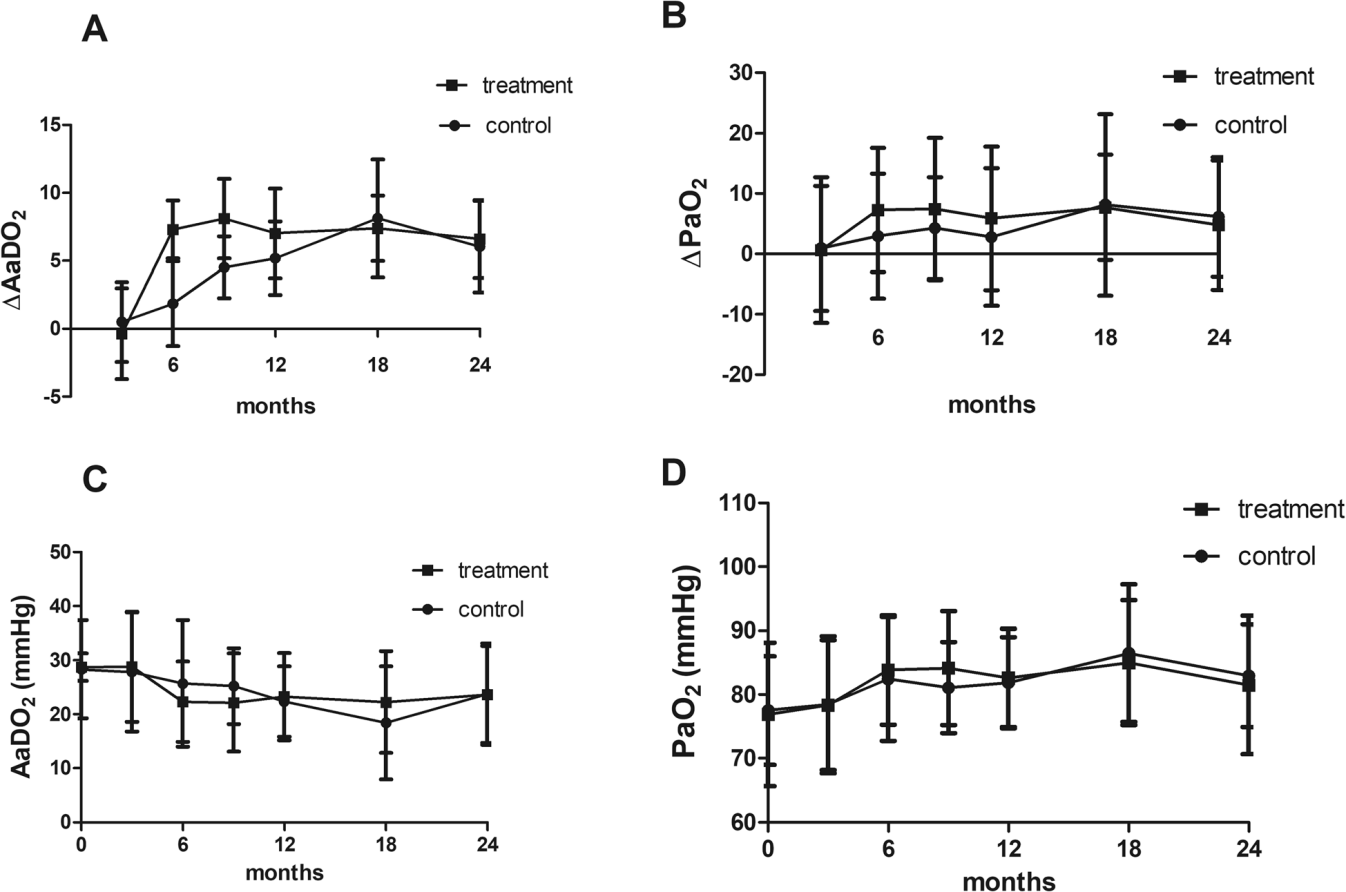
No significant differences were observed between the treatment group and the control group in terms of changes in A-aDO_2_ and PaO_2_ from baseline to 3 and 6 months of treatment and over the following 18 months (**a** and **b**). [A-aDO_2_ levels in control group vs. treatment group: 0.51 ± 12.09 mmHg vs. -0.35 ± 13.76 mmHg, *p* = 0.848 (at 3 months); 1.85 ± 11.21 mmHg vs. 7.31 ± 8.81 mmHg, *p* = 0.146 (at 6 months); 6.05 ± 11.14 mmHg vs. 6.61 ± 10.64 mmHg, *p* = 0.899 (at 24 months)]. No significant differences were observed between the treatment group and the control group for the absolute value of A-aDO_2_ and PaO_2_ during both the treatment period, as well as the follow up period (**c** and **d**)

There were no significant differences between the treatment group and control group based on primary endpoints measured by the change of A-aDO_2_ from baseline to 3 and 6 months treatment and during the following 18 months [control group vs. treatment group: 0.51 ± 12.09 mmHg vs. -0.35 ± 13.76 mmHg, *p* = 0.848 (at 3 months); 1.85 ± 11.21 mmHg vs. 7.31 ± 8.81 mmHg, *p* = 0.146 (at 6 months); 6.05 ± 11.14 mmHg vs. 6.61 ± 10.64 mmHg, *p* = 0.899 (at 24 months)] (Fig. 3a). The change of PaO_2_ level from baseline to 3 and 6 months treatment, and during the following 18 months also showed no significant difference between the two groups (Fig. 3b). The actual level of A-aDO_2_ and PaO_2_ showed no differences during both the treatment period and follow up period as well (Fig. 3c and d) (Tables 2 and 3).

**Table 2:** The clinical parameter of the effects of inhaled GM-CSF during the 6 months treatment periods

	3 months			6 months		
	Control group (n = 17)	Treatment group (n = 17)	*P* value	Control group (n = 13)	Treatment group (n = 17)	*P* value
ΔA-aDO_2_ (mmHg)	0.51 ± 12.09	−0.35 ± 13.76	0.848	1.85 ± 11.21	7.31 ± 8.81	0.146
A-aDO_2_ (mmHg)	27.81 ± 11.04	28.79 ± 10.19	0.794	25.69 ± 11.70	22.31 ± 7.45	0.342
ΔPaO_2_ (mmHg)	0.92 ± 10.34	0.62 ± 12.07	0.94	2.95 ± 10.34	7.26 ± 10.29	0.267
PaO_2_ (mmHg)	78.42 ± 10.74	78.37 ± 10.17	0.988	82.42 ± 9.71	83.84 ± 8.57	0.589
FVC pred (%)	77.24 ± 14.91	78.48 ± 13.72	0.801	80.41 ± 15.60	77.34 ± 23.32	0.688
TLC pred (%)	74.09 ± 11.37	73.12 ± 15.29	0.836	74.28 ± 11.18	78.48 ± 8.88	0.269
DLCO pred (%)	67.12 ± 14.72	69.19 ± 19.83	0.732	70.83 ± 14.62	74.91 ± 14.80	0.465
DLCO/VA pred (%)	95.67 ± 17.32	98.71 ± 21.03	0.641	98.92 ± 12.47	95.93 ± 15.44	0.577
SGRQ symptom	24.84 ± 17.33	24.22 ± 23.32	0.521	29.50 ± 18.61	18.47 ± 19.29	0.097
SGRQ activity	33.45 ± 19.35	24.31 ± 18.92	0.173	28.98 ± 18.78	19.41 ± 17.10	0.149
SGRQ effect	16.38 ± 15.94	17.11 ± 17.86	0.336	21.58 ± 17.60	9.29 ± 10.73	0.023
SGRQ total	14.76 ± 14.52	20.45 ± 17.55	0.285	25.11 ± 16.36	13.88 ± 10.91	0.030
6MWD	494.06 ± 75.43	496.41 ± 75.43	0.926	475.09 ± 85.31	501.13 ± 88.31	0.452
Mean lung density	NA	NA		− 739.64 ± 82.70	− 733.17 ± 61.41	0.804
Total lung volume (ml)	NA	NA		4485.71 ± 971.37	4365.67 ± 1322.58	0.808
Hb (g/dL)	15.42 ± 1.53	15.34 ± 1.42	0.863	15.65 ± 1.79	15.70 ± 1.60	0.932
HCT (%)	44.35 ± 3.54	44.52 ± 3.48	0.881	45.10 ± 4.04	45.50 ± 4.46	0.792
LDH (U/L)	226.88 ± 46.22	223.86 ± 59.32	0.867	230.36 ± 28.96	203.38 ± 60.36	0.130
CEA (U/L)	3.72 ± 3.31	4.87 ± 3.68	0.619	4.40 ± 2.25	3.14 ± 1.74	0.076

Abbreviations: See Table 1

**Table 3:** The primary end point results in the 18 months observational periods after 6 months inhaled GM-CSF treatment

	9 months			12 months			18 months			24 months		
	Control group (n = 12)	Treatment group (*n* = 14)	*P* value	Control group (n = 13)	Treatment group (n = 14)	*P* value	Control group (*n* = 12)	Treatment group (n = 13)	*P* value	Control group (*n* = 11)	Treatment group (*n* = 15)	*P* value
Δ A-aDO_2_ (mmHg)	4.53 ± 7.59	8.11 ± 10.89	0.461	5.19 ± 9.72	7.02 ± 12.35	0.674	8.13 ± 15.02	7.39 ± 8.677	0.579	6.05 ± 11.14	6.61 ± 10.64	0.899
A-aDO_2_ (mmHg)	25.24 ± 7.05	22.16 ± 9.09	0.366	22.35 ± 6.54	23.25 ± 8.10	0.756	18.38 ± 10.50	22.24 ± 9.43	0.343	23.72 ± 9.41	23.66 ± 8.96	0.988
Δ PaO_2_ (mmHg)	4.29 ± 8.38	7.45 ± 11.82	0.267	2.78 ± 11.41	5.89 ± 11.91	0.496	8.13 ± 15.02	7.71 ± 8.72	0.931	6.16 ± 9.88	4.76 ± 10.72	0.741
PaO_2_ (mmHg)	81.08 ± 7.14	84.15 ± 8.93	0.362	81.84 ±7.13	82.59 ± 7.70	0.794	86.48 ± 10.75	84.99 ± 9.76	0.721	82.95 ± 8.05	81.46 ± 10.84	0.707

Abbreviations: see Table 1

### The diffusion capacity and total lung capacity were improved by the end of study

**Fig. 4:**
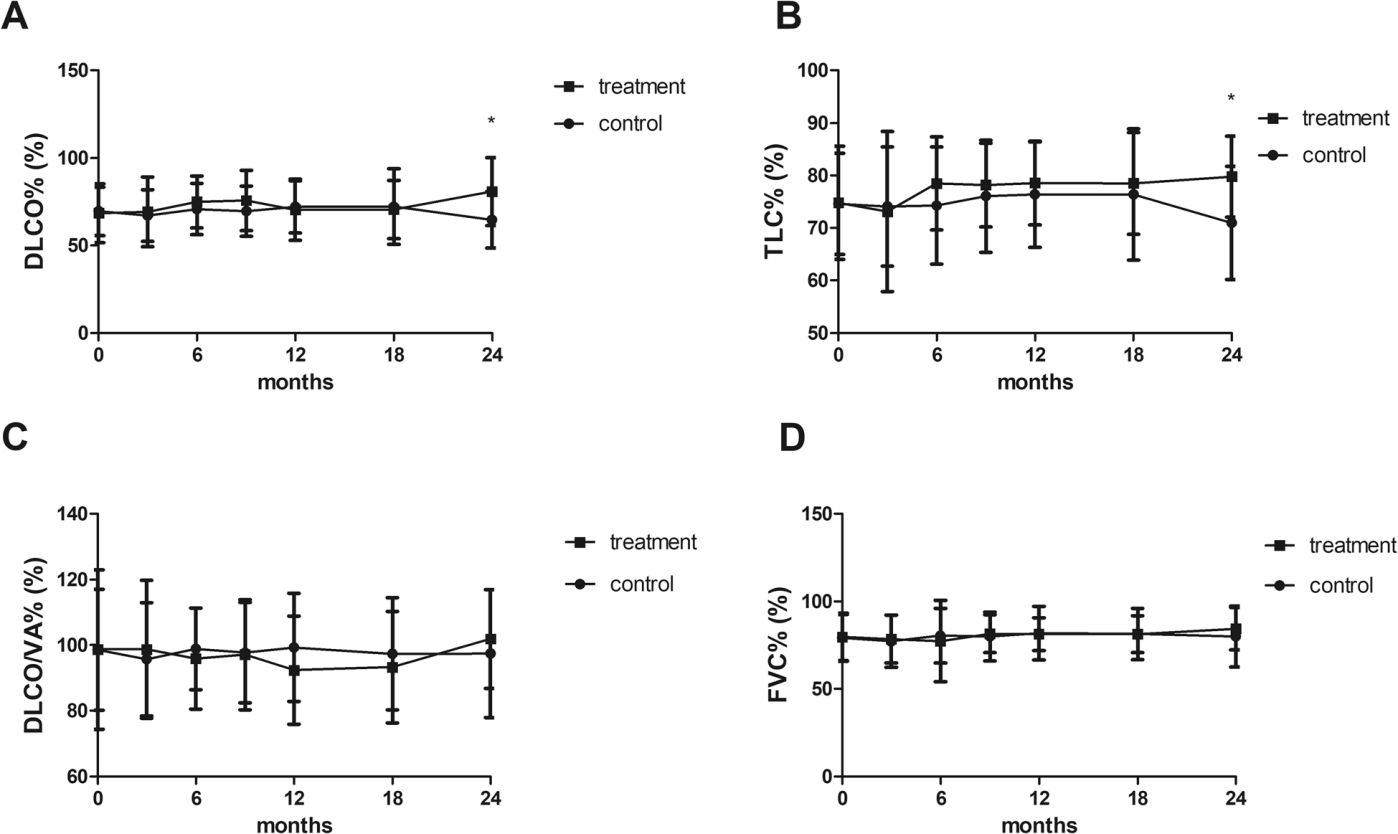
There were significant differences in the DLCO% and TLC% between the treatment group compared to the control group at the end of the study (*P* < 0.05, respectively, **a** and **b**). However, there were no significant differences between the two groups in terms of other pulmonary function tests, including FVC and DLCO/VA, both during the 6-month treatment period and the 18-month follow-up period (**c** and **d**). [DLCO% (control group vs. treatment group): 67.12 ± 14.72 vs. 69.19 ± 19.83, *p* = 0.732 (at 3 months); 70.83 ± 14.62 vs. 74.91 ± 14.80, *p* = 0.465 (at 6 months); 64.67 ± 16.22 vs. 80.87 ± 19.40, *p* = 0.027 (at 24 months)]. [TLC% (control group vs. treatment group): 74.09 ± 11.37 vs. 73.12 ± 15.29, *p* = 0.836 (at 3 months); 74.28 ± 11.18 vs. 78.48 ± 8.88, *p* = 0.269 (at 6 months); 70.97 ± 10.79 vs. 79.77 ± 7.76, *p* = 0.010 (at 24 months)]

Significant differences in DLCO% and TLC% between the treatment group and the control group emerged by the end of the study (Fig. 4a and b). [DLCO% (control group vs. treatment group): 67.12 ± 14.72 vs. 69.19 ± 19.83, *p* = 0.732 (at 3 months); 70.83 ± 14.62 vs. 74.91 ± 14.80, *p* = 0.465 (at 6 months); 64.67 ± 16.22 vs. 80.87 ± 19.40, *p* = 0.027 (at 24 months)]. [TLC% (control group vs. treatment group): 74.09 ± 11.37 vs. 73.12 ± 15.29, *p* = 0.836 (3 months); 74.28 ± 11.18 vs. 78.48 ± 8.88, *p* = 0.269 (6 months); 70.97 ± 10.79 vs. 79.77 ± 7.76, *p* = 0.010 (24 months)]. However, there were no significant differences between the treatment group and the control group in terms of other pulmonary function tests, including FVC, FEV_1_ (data not shown) and DLCO/VA, both during the 6-month treatment period and the 18-month follow-up period (Fig. 4c and d) (Table 2 and supplemental table 1).

### The SGRQ scores increased after 3 months and 6 months of inhaled GM-CSF treatment and 18 months follow-up

**Fig. 5:**
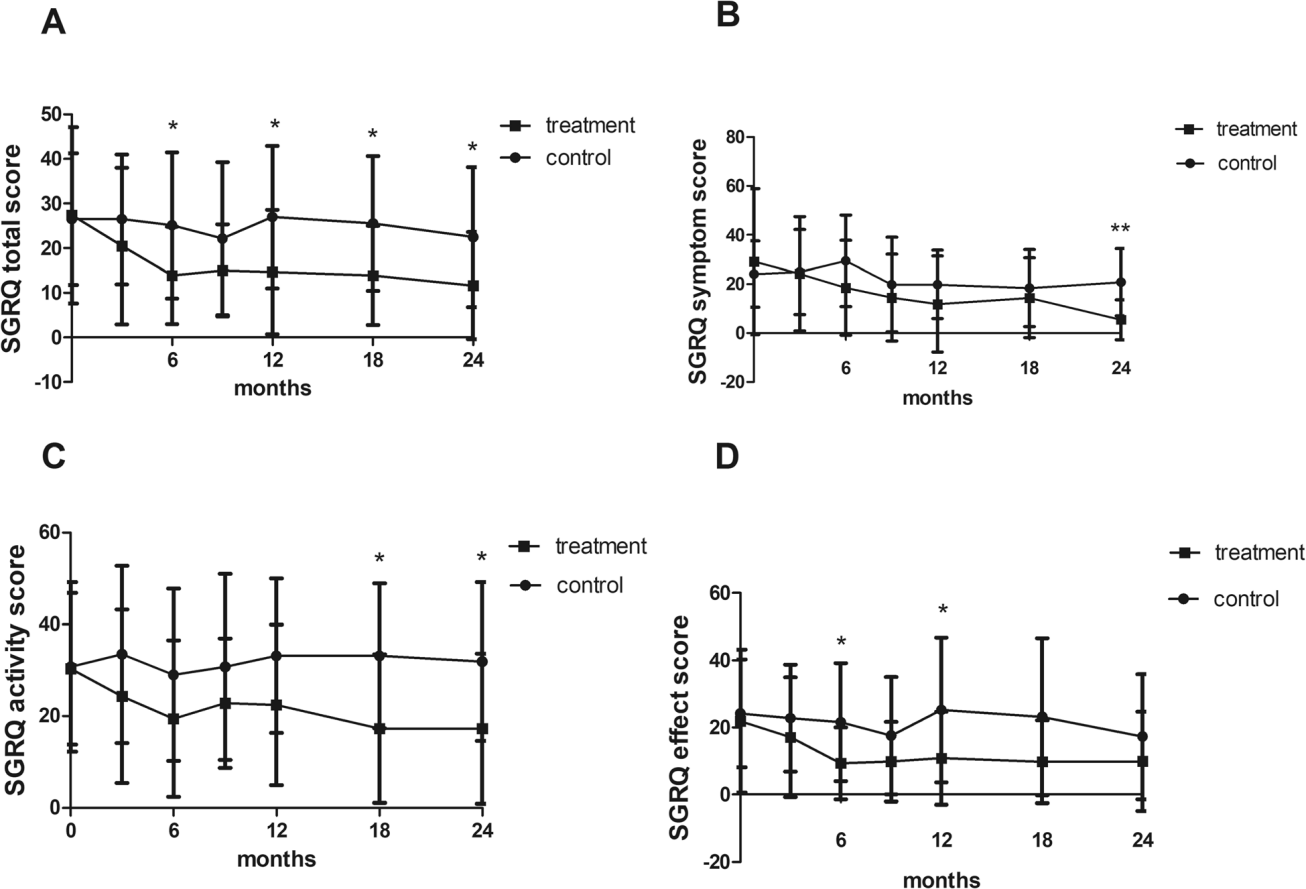
The SGRQ score during the 24-month study period. **a**: total SGRQ score; **b**: SGRQ symptom score; **c**: SGRQ activity score; **d**: SGRQ effect score. *:P < 0.05, **: *P* < 0.01

Meanwhile, we can see obvious differences in patients’ quality of life between the treatment group and control group, as measured by SGRQ. Total SGRQ scores in patients in the treatment group was improved after 6 months of GM-CSF treatment compared to the no treatment group, and the benefits were nearly continuously maintained throughout the 18-month observation period. Similar trends can be observed in symptom score, activity score and effect score, although significant differences between the two groups were not observed at all times (Fig. 5).

### Quantitative CT did not find difference after treatment

There were no significant differences in total lung volume and mean lung density between the treatment group and control group (Table 2 and supplemental Table 1).

### Time to rescue therapy during the 24-month study was not improved

**Fig. 6:**
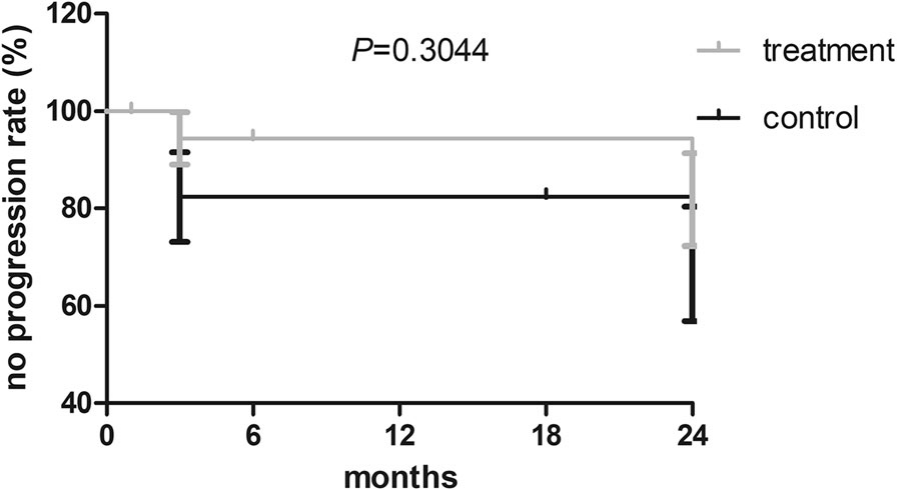
There was no significant difference between the treatment and control groups in terms of no disease progression rate (measured by time to rescue therapy) over the 24 month duration of the study. *P* > 0.05

There was no significant difference in time to rescue therapy between the treatment group and control group. Kaplan-Meier Curve analysis for the two groups was shown in Fig. 6 (*P* = 0.304).

### Safety and tolerability of inhaled GM-CSF

None of our patients died during the trial. None of the patients in the treatment group complained of fever, wheezing or coughing over the duration of their inhaled GM-CSF treatment.

An increase in transaminase levels during GM-CSF inhalation treatment was observed (*P* = 0.037). Fortunately, none of the patients required medical intervention. The highest observed level of transaminase in the GM-CSF treatment group was 123 U/L for alanine aminotransferase (ALT) and 63 U/L for aspartate aminotransferase (AST), while the highest observed level in the control group was 95 U/L for ALT and 77 U/L for AST respectively. All parameters remained stable or gradually declined after patients ceased alcohol consumption and stopped taking medications with possible interfering effects. Peripheral blood neutrophils levels were not obviously increased within the GM-CSF inhalation group when compared to the control group (*P* = 0.429) (Table 4).

**Table 4:** The side-effect of patients with aPAP during the GM-CSF treatment and follow up period

	Treatment periods			Follow up periods		
	No treatment group (n = 17)	Treatment group (n = 19)	*P* value	No treatment group (n = 12)	Treatment group (*n* = 16)	*P* value
Leukocytosis	1/17 (5.9%)	1/19 (5.3%)	1.0^a^	1/12 (8.3%)	0/16 (0)	0.429^a^
Increase in aminotransferases	4/17 (23.5%)	11/19 (57.9%)	0.037	6/12 (50.0%)	4/16 (25.0%)	0.333^b^
Increase in bilirubin	6/17 (35.3%)	2/19 (10.5%)	0.167^b^	4/12 (33.3%)	0/16 (0)	0.051^b^

^a^: *Fisher X*^*2*^ test; ^b^: continuous correction *X*^*2*^ test

No other significant safety and tolerability differences were observed between the two groups during the study.

Other details of side effects that occurred during the study can be found in the supplementary data.

## Discussion

In this study, we prospectively evaluated the effects of inhaled GM-CSF on mild-to-moderate autoimmune pulmonary alveolar proteinosis (aPAP) patients. In contrast to previous reports, no obvious effects were found in our study. During the 6 months of treatment and 18 month of subsequent observation, the primary endpoint, A-aDO_2_ remained unchanged. Health-related quality of life, measured using SGRQ improved after 3 months of treatment, with these improvements and maintained to 24 months. Marginal improvement was also noted in terms of TLC and DLCO by the end of the study. This research provides valuable clinical data and experience for inhaled GM-CSF treatment in aPAP patients who do not meet the criteria for WLL.

The current therapy for PAP patients involves the physical removal of surfactant using a procedure in which the lungs are repeatedly filled with saline and emptied – WLL – which is invasive, inefficient, and is not widely available. Some authors reported that fever, hypoxemia, fluid leakage and other complications occurred in patients treated with WLL [13]. Additionally, the median time to next WLL is around 15 months [14], and about 30–57.6% of patients require further therapy after their first WLL [15, 16]. Though there is currently no consensus, most physicians believe that patients with PaO_2_ less than 70 mmHg when breathing room air or an alveolar-arterial [A-a] oxygen gradient of more than 40 mmHg, or patients with disease progression should receive WLL as treatment [13]. In a cohort study from our center, 33% of patients are stable or experience spontaneous remission [16], while the spontaneous remission rate varies from 8 to 18% in different reports [11, 14, 16, 17]. Considering the occurrence of spontaneous remission in some patients, it becomes a critical question whether GM-CSF inhalation could become a primary treatment for mild to moderate aPAP patients.

After GM-CSF was confirmed to play an important role in the disease mechanism of aPAP, the efficacy of exogenous GM-CSF replacement was assessed in a previous paper. The response rate to this treatment varied, with the efficacy rate being between 62 and 100% when using inhaled GM-CSF [5, 11, 18] while the efficacy rate was 43–75% when using subcutaneously administered GM-CSF [19, 20]. Due to its better responsiveness and tolerance, the use of inhaled GM-CSF is generally recommended [4].

In previous studies, inhaled GM-CSF treatment was prescribed to patients with moderate to severe disease [9, 21–24], with the mean PaO_2_ level in a large prospective study of inhaled GM-CSF treatment on aPAP patients being 61.7 ± 1.4 mmHg [11]. During the preparation of our manuscript, a randomized placebo-controlled study of inhaled GM-CSF was published, with A-aDO_2_ and CT density quantitative measurement being significantly improved, though they concluded that clinical benefits were not significant [25]. There are two major differences in design between our study and Tazawa et al’s study as follows: (1) Tazawa et al. recruited patients with PaO_2_ of less than 70 mmHg (or less than 75 mmHg with symptoms), with the average PaO_2_ being 66.4 ± 8.66 mmHg and 68.8 ± 8.96 mmHg in the GM-CSF group and control group respectively. Meanwhile, we recruited patients with a DSS of between 1 and 3 inclusive, 11/19 from our GM-CSF group and 13/17 from our control group had a PaO_2_ of over 70 mmHg, with an average PaO_2_ of 77.51 ± 8.53 mmHg in the GM-CSF group and 76.88 ± 11.23 mmHg in the control group respectively. (2) While both trials used 6-months of treatment, Tazawa et al. used 125 μg bid continuously, while we used 150 μg bid for 3 months and then 150 μg qd for 3 months. We found no significant response in the primary endpoint as measured by A-aDO_2_. The reasons for the lack of response in our group may be related to the relatively good baseline oxygen content levels in our patients, making changes of this indicator less obvious. Based on our study and on previous papers, the benefits of GM-CSF treatment for aPAP patients with a PaO_2_ of over 70 mmHg (DSS 1 and 2) may be very limited. There were not enough DSS3 cases (PaO_2_ 60–70 mmHg) in our study for subgroup analysis. However, Tazawa, et al. has answered this question with a randomized placebo-controlled study. We believe GM-CSF could be beneficial for those with a PaO_2_ of less than 70 mmHg. Additionally and critically, a significant difference in DLCO, which is a relatively reliable parameter to track improvement in aPAP patients, was observed after 24 months. This suggests that inhaled GM-CSF should be provided to patients in a personally-tailored manner, providing the drug for as long as is necessary for patients to respond to the drug [22, 26]. Longer treatment periods may be necessary for future clinical trials to confirm the efficacy of the inhaled GM-CSF in aPAP over the long term.

We found that inhaled GM-CSF therapy is a well-tolerated choice for aPAP patients as previous studies showed [9, 11, 21, 22]. Although more than half of our patients in the GM-CSF group were found to have slight increases in amino-transferase levels, and a number of abnormal liver function results were observed in the GM-CSF treatment group, the increases in transaminase levels were all slight and no medical intervention was needed for any of the patients. All patients remained in stable condition or gradually improved after the cessation of alcohol and stopping intake of possibly related combination medicines. Therefore, inhaled GM-CSF therapy is a safe and convenient choice for patients.

Our research has some limitations. Firstly, the sample size of the study was small, and there were not enough DSS3 patients for analysis. Our estimated target sample size was based on prior results, done using a patient population with greater disease severity [11], which may have caused an underestimation of the sample size actually needed. Secondly, more patients from the control group, compared to patients from the GM-CSF group, dropped out of the study during the observation period, which may have affected the evaluation of effectiveness when comparing these two groups. Thirdly, the patients in our study did not receive a tailored dosage of GM-CSF treatment, nor did they receive prolonged therapy after the initial 6 months of treatment, which may have caused some latent responders, requiring higher dosages or longer treatment time for a positive response, to remain hidden.

## Conclusions

Six months of inhaled GM-CSF treatment had no effects on the alveolar–arterial oxygen gradient in patients with mild to moderate pulmonary alveolar proteinosis. At the dosage we used, there were changes in some clinical or laboratory measures, but no clinically important changes were noted at the end of study. Our study is an important complement for efficacy in aPAP patients with mild to moderate disease severity.

## Supplementary information

**Additional file 1: Supplemental Table 1.** The secondary end point of clinical parameter changes in the 18 months observational periods after 6 months inhaled GM-CSF treatment.

## Data Availability

The data of our patients is available in the department of medical records at PUMCH and the Affiliated Drum Tower Hospital of Nanjing University Medical School. This data can be released with the agreement of the patients and is available from the corresponding author upon reasonable request.

## Abbreviations

A-aDO_2_: Alveolar arterial oxygen gradient
ABG: Arterial blood gas analysis
ALT: Alanine aminotransferase
aPAP: Autoimmune pulmonary alveolar proteinosis
AST: Aspartate aminotransferase
BAL: Bronchoalveolar lavage
CT: Computed tomography
DLCO: Diffusing capacity for carbon monoxide
DLCO/VA: Diffusing capacity for carbon monoxide corrected for alveolar volume
DLCO%: Percentage of diffusion capacity predicted
DSS: Disease severity score
FEV_1_: Forced expiratory volume in the first second
FVC: Forced vital capacity
GGO: Ground-glass opacities
GM-CSF: Granulocyte macrophage colony stimulating factor
HRCT: High-resolution computed tomography
PaO_2_: Arterial oxygen partial pressure
PUMCH: Peking Union Medical College Hospital
SGRQ: St Grorge Respiratory questionnaire
TBLB: Transbronchial lung biopsy
TLC%: Percentage of total lung capacity predicted
WLL: Whole lung lavage
6MWD: 6 min walking distance
