# Supplementary material for: Inhaled granulocyte-macrophage colony stimulating factor for mild-to-moderate autoimmune pulmonary alveolar proteinosis - a six month phase II randomized study with 24 months of follow-up
Source: Orphanet J Rare Dis. 2020 Jul 2;15:174. doi: 10.1186/s13023-020-01450-4 (PMC7330972; doi:10.1186/s13023-020-01450-4)
Supplement: Supplementary file 1 — Additional file 1: Supplemental Table 1. The secondary end point of clinical parameter changes in the 18 months observational periods after 6 months inhaled GM-CSF treatment. [file 13023_2020_1450_MOESM1_ESM.docx]

**Supplemental data**

**Table 1** The secondary end point of clinical parameter changes in the 18 months observational periods after 6 months inhaled GM-CSF treatment.

|  | The 9^th^ months | | | The 12^th^ months | | | The 18^th^ months | | | The 24^th^ months | | |
| --- | --- | --- | --- | --- | --- | --- | --- | --- | --- | --- | --- | --- |
|  | Control group (n=12) | Treatment group (n=14) | *P* value | Control group (n=13) | Treatment group (n=14) | *P* value | Control group (n=12) | Treatment group (n=13) | *P* value | Control group (n=11) | Treatment group (n=15) | *P* value |
| FVC (L) | 3.18±0.75 | 3.42±0.84 | 0.459 | 3.26±0.77 | 3.38±0.80 | 0.699 | 3.14±0.70 | 3.35±0.83 | 0.497 | 3.20±0.72 | 3.40±0.77 | 0.497 |
| FVC pred (%) | 79.97±14.03 | 81.58±10.87 | 0.744 | 81.86±15.29 | 81.28±9.47 | 0.905 | 81.38±14.73 | 81.28±10.54 | 0.985 | 79.97±17.39 | 84.43±12.24 | 0.327 |
| TLC (L) | 4.69±0.95 | 4.87±1.07 | 0.644 | 4.78±0.98 | 4.88±1.03 | 0.787 | 4.66±0.96 | 4.86±1.13 | 0.638 | 4.49±0.92 | 4.91±0.91 | 0.205 |
| TLC pred (%) | 76.04±10.74 | 78.18±8.00 | 0.567 | 76.38±10.07 | 78.56±7.97 | 0.538 | 76.40±12.50 | 78.51±9.72 | 0.641 | 70.97±10.79 | 79.77±7.76 | 0.010 |
| DLCO pred (%) | 69.59±14.35 | 75.66±  17.23 | 0.344 | 72.08  ±14.94 | 70.41±17.49 | 0.794 | 72.23±21.66 | 70.55±16.62 | 0.829 | 64.67±16.22 | 80.87±19.40 | 0.027 |
| DLCO/VA pred (%) | 97.68±15.33 | 97.08±16.81 | 0.925 | 99.28±16.54 | 92.37±16.48 | 0.287 | 97.35±17.13 | 93.28±17.00 | 0.558 | 97.42±19.54 | 101.92±15.08 | 0.705 |
| SGRQ symptom | 19.83±19.29 | 14.49±17.76 | 0.470 | 19.79±13.96 | 11.81±19.56 | 0.237 | 18.36±15.77 | 14.41±16.21 | 0.534 | 20.80±13.63 | 5.42±8.17 | 0.001 |
| SGRQ activity | 30.70±20.28 | 22.81±14.12 | 0.255 | 33.12±16.87 | 22.43±17.51 | 0.119 | 33.14±15.80 | 17.28±16.20 | 0.019 | 31.86±17.29 | 17.27±16.38 | 0.030 |
| SGRQ effect | 17.56±17.50 | 9.86±11.87 | 0.196 | 25.20±21.58 | 10.86±13.84 | 0.049 | 23.17±23.39 | 9.80±12.28 | 0.081 | 17.2723±18.66 | 9.92±14.76 | 0.255 |
| SGRQ total | 22.10±17.11 | 15.02±10.28 | 0.206 | 26.97±15.95 | 14.65±13.92 | 0.042 | 25.52±15.12 | 13.89±11.11 | 0.035 | 22.48±15.63 | 11.61±12.00 | 0.048 |
| 6MWD | 456.82±58.99 | 491.71±73.63 | 0.213 | 474.38±55.42 | 473.53±52.62 | 0.968 | 497.92±122.73 | 503.69±74.36 | 0.887 | 486.09±68.78 | 506.13±77.41 | 0.501 |
| Mean lung density | NA | NA |  | -757.67±55.24 | -736.67±55.79 | 0.364 | -755.30±45.49 | -739.17±53.92 | 0.463 | -755.00±62.96 | -737.33±53.95 | 0.477 |
| Total lung volume (ml) | NA | NA |  | 4678.83±1017.86 | 4241.33±1229.30 | 0.353 | 4563.50±85.65 | 4482.00±1242.13 | 0.873 | 4640.55±958.69 | 4581.08±1107.37 | 0.892 |
| Hb (g/L) | 156.91±16.48 | 160.88±14.35 | 0.512 | 156.93±15.32 | 158.76±16.77 | 0.512 | 156.21±17.44 | 159.94±16.81 | 0.551 | 160.85±17.46 | 158.81±16.15 | 0.748 |
| HCT (%) | 44.95±4.29 | 46.02±3.97 | 0.512 | 44.95±4.29 | 46.02±3.97 | 0.510 | 45.69±4.94 | 43.37±11.49 | 0.485 | 40.91±17.16 | 46.46±4.30 | 0.221 |
| LDH (U/L) | 228.00±38.34 | 212.41±41.80 | 0.329 | 217.54±39.61 | 226.94±38.69 | 0.519 | 221.14±53.11 | 208.52±36.41 | 0.440 | 265.46±66.48 | 233.67±29.39 | 0.127 |
| CEA (U/L) | 3.99±2.57 | 3.65±1.37 | 0.665 | 4.05±2.71 | 3.62±1.65 | 0.588 | 3.95±2.63 | 3.95±2.74 | 0.995 | 5.17±2.88 | 3.87±2.98 | 0.238 |

***Supplementary of safety and tolerability of inhaled GM-CSF***

One patient in the control group was removed from the study on the basis of requiring rescue therapy, and he reported fever (maximum temperature is 38.8°C) and myalgia during the first week after he inhaled 150µg GM-CSF twice daily. His symptoms were relieved with ibuprofen. After that, no fever, myalgia nor any other symptoms occurred during the subsequent GM-CSF treatment period.

One patient in the GM-CSF treatment group was admitted to hospital 9 months after enrollment because of proteinuria and was diagnosed with obesity-related nephropathy confirmed by renal biopsy. The investigator concluded that the event was not related to the GM-CSF inhalation treatment.

One 27-year-old female patient was diagnosed with asthma 3 months after completing her inhaled GM-CSF therapy. Her first asthmatic attack occurred 1 month after her last dosage of GM-CSF, so the investigator concluded that the situation was not related to the GM-CSF inhalation treatment.
